
JOURNAL INFORMATION
==============================
NLM Title Abbreviation: Med Chir Rev
NLM Journal ID: 0034617

The Medico-Chirurgical Review

Publisher: Biomedical Journal Digitization Project

ARTICLE INFORMATION
==============================
PMCID: PMC4966183
PMID: 29917605
Article version: 1
Subjects: Articles

Hospital Practice

Print publication date: 1828 Jul 1
Volume: 9
Issue: 17
First page: 158
Last page: 168
1828 Apr 12
License: This work is free of known copyright restrictions. For more information, please see PMC Back Issue Digitization.
License URL: https://creativecommons.org/publicdomain/mark/1.0/

==============================

### HOSPITAL PRACTICE.

### ST. GEORGE'S HOSPITAL.

### 14. OSSIFICATION OF THE CORONARY ARTERIES.

In the 6th Fasciculus of our last number, we took up the subject of angina pectoris, and alluded to the occasional, and, indeed, rather common occurrence of ossification of the coronary arteries in that disease. A short time ago a curious case occurred at St. George's Hospital, which afforded a good specimen of the pathological change in question, and was the occasion of a slight disagreement amongst the doctors.

W. Jones, æt. 72, was admitted, March 7th, under the care of Mr. Brodie, labouring under retention of urine. He stated that he had suffered from difficulty of making water, requiring the occasional introduction of the catheter, for the last ten years or more, and that he had recently been a patient in Bartholomew's Hospital, where Mr. Lawrence laid him on his belly, introduced his finger up the anus, and "broke some of the small bones of his back"!! On examining the rectum for a confirmation of this terrible tale, there was found a pretty voluminous cluster of common *piles*, some of which were in a state of ulceration, but no further evidence of Mr. Lawrence's shocking cruelty. A catheter was introduced, though with some difficulty, in consequence of a considerable enlargement of the prostate gland, and a quantity of bloody urine evacuated. The instrument was introduced regularly three or four times a day—the bowels opened, and suppositories of opium and conium administered to allay the extreme pain which the patient experienced in making water. On the morning of the 14th, he went to the necessary, returned, got into bed and expired almost immediately. He had never made a complaint of vertigo or of being subject to spasms of any kind, but his countenance was unhealthy, and he appeared to have drunk freely.

*Dissection.* The bladder was much thickened, and its mucous coat rough and granular; the prostate was exceedingly enlarged, and its middle lobe projected within the neck of the bladder, the size of a hazel nut. There were two calculi in the bladder; a great many prostatic calculi; and half a dozen false passages, of various directions and dimensions, some leading into the prostate, and some through it. On examining the heart, it was found *enlarged and very flabby*, whilst its coronary arteries afforded a good specimen of the ætheromatous and calcareous depositions within their coats. Besides this, there were full two ounces of serum in the ventricles of the brain.

*Remarks.* Thus far our reporter. He has not seen quite so many cases of this kind as ourselves. The large and flabby state of the heart was far more likely to be the cause of sudden death in this instance, than the calcareous depositions in the coats of the coronary arteries.

### 15. CURIOUS MALIGNANT TUMOUR GROWING FROM THE VAGINA.*

E. B. æt. 23, after a fall, noticed a small tumour on the right side of the vagina, which gradually increased, giving her, at times, very considerable pain. She made no mention of the disease until it had existed for nearly two years, when she entered St. George's Hospital, under the care of Mr. Brodie. This was in the month of February last, and she then presented the following appearances:—From the anterior part of the vagina, just below the clitoris, there arises an odd-looking, circular tumour, about the size of a walnut. It pushes the orifice of the meatus urinarius to the left side—its lower half is encircled by a prolongation of the vascular lining membrane of the vagina; whilst its upper is rough, of a yellowish-brown hue, and sloughy appearance, having evidently protruded through the lining membrane, as the fungus arising after chronic inflammation of the testicle does through the ulcerated scrotum. The tumour was so circumscribed, that Mr. B. imagined he could get his finger in some measure behind its root; the os tincæ, and other parts were sound.

She was kept quiet, in the hospital,

* London Med. Gazette, No. 16.

for nearly a month, at the end of which time the tumour had decidedly increased in size, and put on a more sloughy appearance. Under these circumstances, it was thought adviseable to have recourse to that *dernier resort* in such cases, the knife, and on the 11th March, the operation was performed by dissecting off the vaginal membrane from the base of the tumour, and then completing its separation from the parts beneath, with the handle of the scalpel. This was accomplished with much ease, care being taken to avoid injury to the urethra, and on making a section of the diseased mass, after its removal, it was found to present a malignant appearance, although neither true scirrhus nor fungus hæmatodes. Bleeding took place from a vessel at the bottom of the wound, when the patient had become warm in bed, so that it was necessary to apply a ligature, and pledgets of blue lint. These means were effectual in arresting the hæmorrhage, and the patient at present is going on very well.

In the report which contains the above, there is related a case of "preternatural abdominal pulsation." The patient is affected with disease of the spine, the lower dorsal vertebræ projecting backwards, and the aorta can be felt pulsating very strongly at the umbilicus. At one time a distinct tumour was felt in this situation, possessing most decidedly the aneurismal thrill, and having many of the characters of an aneurismal dilatation of the vessel. As the affection of the spine improved, this tumour disappeared, and at present the aorta is felt beating of its natural calibre. The reporter imagines that the curve *backwards* in the dorsal vertebræ, will naturally induce, as indeed always happens, a corresponding curve *forwards* in the lumbar vertebræ, in order that the centre of gravity may fall within the base of the column. This unusual convexity of the loins will of course render the abdominal aorta more prominent than usual, and this accounts for the pulsation which it presents.

---

### MIDDLESEX HOSPITAL.*

### 16. CASE OF HERNIA, WITH OBSERVATIONS AND ORIGINAL ILLUSTRATIONS.

A man, æt. 47, who had been subject to the disease for twenty years, was admitted into the Middlesex Hospital on the 10th February, with scrotal hernia on the right side, which could be readily reduced within the ring. It appeared that the gut had not descended for five years previously until the evening of the 7th, when he felt sick and vomited. On the 8th, he applied to a druggist, who attempted ineffectually to reduce it, but a surgeon who saw him in the afternoon, succeeded in removing the tumour, so that he could push his finger into the external ring. The patient was relieved, but the bowels could not be moved, although he took, in all, between the mornings of the 8th and 10th, when he was admitted into the hospital, fifty grains of the ext. col. comp. and sixteen of calomel as well as several enemata. On his admission, there was constant vomiting—tympanitis, fluttering pulse, &c. and he was ordered by the house-surgeon castor oil, laudanum, and a clyster. Three motions were obtained, but the man died in the night. The hernia came down repeatedly during the day, but each time could be readily returned.

*Dissection.* On cutting into the abdomen the intestines were found blown up with gas, and highly inflamed. On opening the sac there was found a knuckle of intestine, the coats of which were more pulpy than natural, and of a dark brown colour, in some places, indeed, perfectly black. The stricture was seated in the neck of the sac, and the gut at this point was soft and apparently ready to ulcerate. Around the neck of the sac the peritoneum was detached from the inside of the abdominal muscles, and hung in a loose fold. It was now evident that the intestine had never been properly reduced, but the sac, with its contents, had been pushed back successively through the outer ring, inguinal canal, and inner ring behind the abdominal muscles, tearing down the connexions between these muscles and the peritoneum, opposite the

* Med. Gazette, No. 16.

internal ring. The description in the report is none of the most lucid, and it is eked out by two wood-cuts, which only serve, after all, to make the *obscurum obscurius.*

We really think that the true state of the case might have been guessed at during life without any very great difficulty. When a hernial tumour, after having been irreducible for many hours, can be pushed up out of sight, but immediately returns, and the symptoms of strangulation continue unabated, the natural conclusion is, that it is the *sac* which is reduced, whilst the stricture still remains. This stricture cannot be in the outer ring, neither can it be in the inner, for the herniary tumour can be readily returned through both; where then can it be but in the *neck* of the *sac?* The conclusion is almost inevitable, and the practice as plain—an operation as soon as possible. We do not believe that any operation would have saved the patient's life at the time he was admitted into the Middlesex, because the symptoms were then so far advanced as to mark general peritoneal inflammation; but we do believe, that were such a case to occur again, the surgeon would be fully warranted in operating, under tolerably favourable circumstances in other respects.

Whilst noticing this case, we may be excused for introducing one or two which we lately had an opportunity of witnessing, where hernia was complicated with hydrocele, rendering the diagnosis, in some measure, obscure. We are aware that there are some folks who say there is no difficulty in the matter, and affirm, that the surgeon who mistakes another affection for hernia, or hernia for any other affection, knows nothing of his profession, and deserves to be cashiered! It may be all very well to enunciate a proposition like this in a medical society, because it makes the vulgar stare, and the orator is thought a "knowing fellow," but those who are engaged in practice, occasionally find, to their cost, that the sailing is not always so smooth, as these garretteers would lead them to imagine.

A young man was admitted some little time ago into St. George's Hospital, at about 11 o'clock in the forenoon, with all the symptoms of "acute" strangulated inguinal hernia. There was great anxiety of countenance, a hard but low pulse—sickness—constipation—pain on pressure of the abdomen, all the symptoms, aye, and strongly marked too, which were laid down in the Westminster Society as diagnostic of strangulation.—There was much tension of the tumour, which was large, and appeared to consist of more than a common hernia. The man was half tipsy, and no accurate history could be obtained, save that he had been engaged in a drunken fray in the morning, had received a blow or strain in the scuffle, and had immediately perceived the swelling in the groin. He was bled, purged, put into the warm-bath, and the taxis employed without the slightest effect, so that it was determined to operate at 4, P. M. In the mean time, however, cold, by means of the sulphuric ether, was employed, and just at four o'clock, when all was ready for the operation, and the faces of the pupils, collected from far and near, were dimpled at the prospect with "smirks and wreathed smiles," the house-surgeon felt something "go up," and the patient declared he was in heaven! A very considerable tumour still remained, and it ultimately turned out that the patient had for some time been affected with a clap, and that the tumour was caused by a combination of common hernia, varicocele, and hernia humoralis! All this was evident enough in the course of a few days, but we would have defied any one, even were he as clever a personage as Mr. Lambert himself, to have distinguished them on the man's admission.

Another case occurred at the same hospital within the last fortnight which is worth recording. A young man, a groom, was admitted with a tumour in the right groin, having all the characters of a strangulated scrotal hernia. It was tense, painful, and stretched from the scrotum up through the outer ring along the inguinal canal to the internal ring. The testicle could be felt below, but there was some degree of enlargement about it, which gave a degree of obscurity to the case. The history which the patient gave was confused and unsatisfactory, but it was evident that he had been subject to the occasional appearance of a tumour in the groin since childhood, that it would remain down for some time, attended with severe symptoms, and then disappear spontaneously. The tumour descended, in the present instance, on the morning of his admission, whilst exercising a horse; he felt sick at the time, afterwards vomited, and the bowels were constipated. The warm bath, bleeding to syncope, and the taxis, were tried in vain; then the sulphuric ether and bags of pounded ice were applied, but these also failed. As a dernier resort, independent of the operation, a tobacco enema was thrown up, and when its effects had shewn themselves on the system, the taxis was employed, and the hernia reduced. Still a tumour remained, evidently a collection of fluid, but where that fluid was situated it was not so easy to determine. The testicle was a good deal wasted, and there was hydrocele of the tunica vaginalis, that was clear—on raising the scrotum, however, or making pressure on it, the fluid, in a great measure, disappeared from the tunica vaginalis, and passed along in the course of the cord, as high as the inner ring. It could not be forced into the abdomen, so that it was not congenital hydrocele, and on coughing, the gut descended in front of it. What the exact nature of the case may be it is difficult to say, but we have detailed it, because, with the former, it proves that hernia, when complicated with hydrocele or enlargement of the testicle or cord, is exceedingly obscure in its history and symptoms, and further, that such complication may give to the hernial tumour an appearance of extent and danger which in itself it really does not possess.

---

### 17. ANATOMY.

We are glad to see that petitions are preparing for Parliament from all parts of the town and country, and that there is every chance of a committee being granted for the purpose of inquiring into the evil and the remedy. In the mean time, we are convinced that nothing is so likely to prejudice the cause as public discussions on the subject in newspapers and other popular periodicals. The crude speculations which are there emitted respecting the remedy for scarcity of subjects, cannot but increase the prejudices which now exist, and engender others not yet in operation. The inquiries made by the committee will elicit all the information that can be desired—and the remedy should flow from this information, and from such suggestions as may be tendered by the witnesses.

In the mean time, we have to draw the attention and the sympathy of the profession to the case of a young medical gentleman, Mr. Davies, an apprentice at the Warrington Dispensary, who, with some others, has been prosecuted, *for having in his possession*, a body that had been disinterred, for the purpose of dissection. The corpse was that of a young woman, a farmer's daughter, and the prosecution appears to have been carried on by a society of Baptists, to which the father belonged. As one great object of the present application to Parliament is to rescind the law that punishes the *possession of dead bodies*—not the *resurrection* of them—and as the trial of this question has cost the parties a large sum of money, and the question is to be again tried in the Court of King's Bench, it behoves the profession to come forward at once, and raise a subscription for Mr. Davies.

An extract from Mr. Brougham's defence will put our readers in possession of the merits of the case, and therefore we shall here insert it.

" Mr. BROUGHAM said his Learned Friend, the Sergeant, had seemed to anticipate, in his very temperate, judicious, and skilful statement, that he (Mr. B.) might deny that there was a law which rendered it an offence indecently to disinter a body. He meant not, however, to deny any such thing. Whilst he said that dissection was necessary for the good of the living,—that there was no other means of saving the life of man from disease and accident than by the examination of bodies after death, he did not deny that exhumation might be carried on in a way to outrage the feelings of the community. But the jury were not to suffer their feelings of delicacy or decency to carry them away, or to make them satisfied on slight evidence that the defendants were the persons engaged in this transaction. The defendants were charged with two several matters: the first, which was variously put in the first two counts of the indictment, was for a conspiracy to disinter indecently, for the purpose of bringing away a certain individual body for dissection: the second, which was contained in the last four counts, was simply for receiving, and having in their keeping, a body for the purpose of dissection, knowing that it had been unlawfully disinterred. It was material to keep these two charges quite distinct in their minds. He should endeavour to show that there was no evidence to convict the defendants, *Davies* and *Hall*, for whom he appeared, of either of these two charges. The second, they would observe, was not the mere having a body for the purpose of dissection; he might have this lawfully: the offence was in having a body, knowing it to have been unlawfully, that is, indecently disinterred. It was an observation applying to this, in common with all charges of conspiracy, that, when it was once proved that the offence had been committed by some one, nothing was so easy as to hook in, as it were, and attach to the body of the evidence, any other person who had had any connexion with, or touching of, the subject from beginning to end. It was hardly possible for any one to approach or speak to, much less to have any dealings with, any individual connected with the subject-matter of the misdemeanor, without furnishing evidence which would hook in a person as a party to the conspiracy, even though the evidence was extremely slight. If, indeed, it had been proved that two persons were seen going to the grave and taking the body, and that as they went, or came away, they were joined by a third person, they would naturally conclude that that person was engaged in the conspiracy. But how different was the present case! It was not pretended that the gentlemen for whom he appeared had ever had the slightest connexion with the persons who went to Hill Cliff. The jury did not know who went there; but suppose, for example, that Box and Ashton had gone, (of which there was not the slightest evidence,) still there was not a title of evidence to connect Davies and Hall, or Blundell, with them. If a person had goods in his possession which he could not account for, and which had been stolen, the inference was strong that he had received them, knowing them to be stolen; but he should show that no such inference arose, or could arise, in the present case, from the particular nature of the traffic. They had evidence from the prosecutor himself, (for Dr. Moss was subpœnaed for the prosecution,) that the common way in which bodies were obtained for dissection was by purchasing them from unknown persons, or without the knowledge of the surgeon as to where the bodies came from. And be it remembered, that, without dissection, anatomical and surgical studies could not be carried on;—that young men carried on these studies in the course of their surgical education;—that professorships of anatomy had been established by the Crown in our Universities;—that those professorships were useless without dissection, and that dissection could not be carried on without a supply of dead bodies. The mere having of bodies, therefore, for the purposes of dissection, was not only lawful, but positively encouraged by law. But the way in which bodies were commonly obtained, was, that a resurrection-man, as those men were called, who procured bodies, no matter how, either by disinterment or by importing them from abroad, came to the surgeons offering a body for sale, and that the body was purchased, bought, and paid for, without the surgeon knowing any thing of the manner in which it was got. This being the case, the inference usually arising from the possession of other kinds of property, was at an end. This fact was of the utmost importance, and on this he would be content to rest the case, for the question was neither more nor less than whether a surgeon should ever, henceforth, be allowed to dissect a subject, or whether surgical science should be acquired. The question, therefore, was, whether there was any thing in the evidence to show that Davies and Hall had not been as ignorant of the way in which this particular body had been obtained, as Dr. Moss was: or whether their own account of the purchase of the body, from a man unknown to them, for four guineas, was not the true one. The account which Hall gave before the magistrates, and which Davies gave to Dr. Moss, perfectly concurred; they both stated that the body was to be brought by a person unknown to them. There was, therefore, an entire disconnexion between the fact of the disinterment, and the having the body. The practice of dissection was never, even in the most barbarous periods of the history of this country, considered illegal. There was, indeed, a law passed in the reign of

James I., of sapient and sacred memory, against having bodies for purposes of witchcraft; and the same statute prohibited a dealing with unclean spirits; (*laughter*,) but this indictment was for the disinterment of a body indecently, and for receiving it, knowing it to be so disinterred. Under any other circumstances, it was not unlawful to have a body for the purpose of dissection; and there was not a tittle of evidence to convict his clients of having obtained the body by unlawful means. If the jury should go so far as to say, under the second charge, that if he took a body into his possession, and dissected upon it, without knowing any thing of the manner in which it was obtained, he was therefore guilty of an offence: let their verdict plainly say so, and let all the frightful consequences follow. But they would say no such thing. Dissection was encouraged, nay, patronized by the law. The body might have been brought from abroad, for any thing the defendants knew: it might have come from a country like France, where surgeons had a right to dissect bodies of persons who died in certain circumstances; or from a country where the bodies of galley slaves were given for dissection, as in the South of Europe, and indeed in France; or from Ireland, whence bodies are constantly imported into this country. The more strongly the disinterment of bodies was prohibited by law in this country, the more he was bound to presume that any body found in a dissecting-room was lawfully obtained. The young men, his clients, were students in surgery—decent, attentive, diligent young men, following the necessary studies of their professions,—and with characters such as they had heard from the witnesses for the prosecution, was it to be presumed that such persons had obtained a subject in an unlawful manner? With these observations he submitted the case to the jury, confidently trusting to their good sense and sagacity in sifting the evidence under these two distinct heads of charge; and he confidently looked to their sound, manly good sense to guard them from such a decision as mere uncontrolled feelings might be calculated to produce."

"Mr. Baron Hullock, in charging the jury, said that, to prove a conspiracy, it was not necessary that all the parties should be shown to have been together, but if, from all the circumstances of their conduct, it was to be presumed that there must have been a previous concert, that would be enough to establish the charge. But as conspiracy was an offence of serious magnitude, they should be satisfied, before finding a verdict of guilty on the former part of the indictment, that the conduct of the defendants was the result of previous concert. There was no evidence against Ashton, who must, therefore, be acquitted. As to Box, the evidence was very slight. If they thought the rest, or any of them, were in possession of the body under circumstances which must have apprized them that it was improperly disinterred, the jury would find them guilty of the latter part of the charge. Blundell's being a stationer did not relieve him from suspicion, for his brother was in the Dispensary. The only bodies legally liable to dissection in this country, were those of persons executed for murder. However necessary it might be, for the purposes of humanity and science, that these things should be done, yet, as long as the law remained as it was at present, the disinterment of bodies for dissection was an offence liable to punishment. The amount of that punishment must always depend on the circumstances of the case. The only evidence against Hall was his own account given to the magistrates; they would judge whether that clearly showed him to have had a guilty knowledge of the way in which the body had been obtained.

"The jury deliberated for a few minutes, and then pronounced Davies and Blundell *Guilty* on the four last counts, which charged a possession of the body, with knowledge of the illegal disinterment; and *Not Guilty* of the charge of conspiracy.

"Hall, Box, and Ashton were *acquitted* of the whole charge.

---

"☞ The moral effect of this trial is to exculpate all the defendants from any thing like conspiracy to procure the disinterment of the body of Jane Fairclough, or any other body. A body had been disinterred, and was offered to certain parties to whom it might be supposed to be useful. Two of the defendants only have been found guilty of receiving a body which had been so previously obtained, and the jury infer a knowledge, on their parts, that it had been illegally disinterred. The mere legal effect, then, of the trial is to raise this point, namely, whether a person obtaining a subject for dissection, under any circumstances in which he cannot be connected with the disinterment, is punishable as having received it, knowing it to have been unlawfully taken from the grave, if it so happens that it has been so taken by persons unknown; or if the defendant cannot prove it to have come from abroad, or to be the body of a person executed for murder in this country. The Court of King's Bench will have to decide, therefore, whether Mr. Davies and Mr. Blundell are guilty, or not, of any offence whatever; and in every point of view the question now raised, and in its present shape, must be highly interesting to the public generally, and to the medical profession particularly. Its decision will be of national importance, if its merits be efficiently argued in the Court above, and will lead to the settlement of the existing, or the adoption of a new, law on the subject."

We earnestly urge our brethren to come forward on this occasion, with sums, however small, in order to defray the expences of the lawsuit past and that which is to come. Having only received the document under consideration just as the last sheet of this fasciculus was going to press, we had not time to solicit subscriptions. But we put down our own, and have no doubt that the example will be quickly followed by others.

SUBSCRIPTION FOR MR. DAVIES.

[The List at Mr. Highley's, Fleet Street.]

| | £ | s. | d. |
|---|---|---|---|
| Medico-Chirurgical Review .. | 5 | 5 | 0 |
| James Johnson, M.D. ...... | 1 | 1 | 0 |

☞ *The names will be published or not according to the wishes of the subscribers.*

---

18. HOSPITAL REPORT IN THE LANCET.

The following protest against a late report in the Lancet and several newspapers is signed by more than 150 students, four-fifths of whom were actual spectators of the operation which has been so misrepresented and falsified by some person or persons, to us, (and we suppose to all others,) at present unknown. We envy not the feelings of the anonymous reporter—nor even those of the editor, who published such a statement, on the faith of some concealed assassin of character.

"ANATOMICAL THEATRE, GUY'S HOSPITAL.

"TO THE EDITOR OF THE MORNING HERALD.

"Sir,—We, the undersigned Dressers and Pupils of the Borough Hospitals, have observed, with mixed feeling of surprise and regret, that in your paper of Saturday last, among the various channels of information, which in your official capacity as Editor you deem it your duty to explore, you should have selected from *The Lancet* of the same date, what purposes to be a report of an operation recently performed at Guy's Hospital:—of surprise, on the one hand, that you had no misgivings as to the truth and accuracy of the publication, no suspicion of the quarter from which your information was derived; and of regret, on the other, that, presuming guilt on such evidence, you should have given a temporary sanction and stability to the statement by inserting it in your highly respectable journal.

"But, Sir, as you did not stop to ask 'are these things so?' before giving them publicity, we at least expect that, in the impartial exercise of your censorship, you will readily repair the mischief your instrumentality has caused, by setting forth in those columns which contained the charge the following unanimous and unequivocal contradiction.

"Indeed, we should be extremely forgetful of the advantages secured to us by this institution, and but ill requite the kindness we have uniformly received, were we to 'spare to speak' at this moment, when the character of one of its officers has been so foully and falsely assailed. Hence we feel we are called upon, in an especial manner, to justify the conduct of that individual; to testify to his qualifications as a teacher, to his superior skill as an operating surgeon, and to his worth and integrity as a man. We therefore formally protest against the defamatory calumnies which have been noised abroad, and avowedly declare that the report of the case, as given in *The Lancet*, is altogether amplified and exaggerated; that there is every distortion of facts, and insertion of what are not facts, to bring the operator into contempt, and to sully his fair character. But, as it would occupy too much space in your valuable columns to *dissect out* and falsify each assertion, we shall content ourselves with affirming, that the protracted time of the operation was attributable to the unusual and extraordinary difficulties which presented themselves; that these difficulties were met and overcome by the operator with coolness; and that he neither used any expressions but those called for by the exigencies of the case, nor spoke in the unguarded manner which has been so maliciously, but ridiculously, imputed to him; and that the last assertion, viz.—of the patient being kept upon the table *bound* whilst the operator was explaining—is only one of the many falsehoods that have been so meanly devised and industriously circulated against him.

"We have been thus brief in our contradiction of the statements, or rather the misstatements of *The Lancet*, because it is notoriously admitted as a fact among the reputable part of the profession, that to quote from *The Lancet* is at all times a ground for doubting the truth of such quotation. Besides, the spirit with which the report is written plainly disproves the sincerity of the production—*mole ruit suâ.*

"But what we have said will suffice to show, that although the best among us cannot escape with impunity, there will always be found those who are ready, humble as their efforts may prove, to expose such personal hostility, and to assure the public that his attacks have the least effect where they are intended to do the most injury."

Here follow the signatures of nearly 200 pupils.

---

From a sensible and energetic letter in the same newspaper, and which is signed ULTOR, we select the following passage. It well deserves the serious attention of our brethren. The day is fast approaching, when the profession in this country will be irretrievably marked out as the scorn of all other classes of society, unless they follow the example set them by the medical students in the Borough, and shew their detestation of the instrument of evil by determining never more to read it or encourage it.

"If, indeed, to tear aside the veil which decent custom has drawn over scenes of bitter trial incidental to our common nature,—to mock the agonies of afflicted humanity by burlesque associations,—to insult the memory of the meek and confiding sufferer now gone, and set his sorrowing relatives on the rack,—if to blast the character of the medical profession, and shake to its centre the public confidence in it,—if these are to be the blessed results of the modern intellectual system, I say, Sir, it is an accursed system, and that it threatens to overturn all that it is our dearest interest to uphold—all that it is the fondest object of social as well as individual man to protect, cherish, and preserve.

"But, Sir, the motives of this hospital chronicler are the opposite of the liberality which he assumes; and this incalculably aggravates his wantonness on the present occasion. He affects to advocate the liberty of the press—he does but abuse it. He claims the patronage of a public which he deceives, by arrogating for himself a bold statement of the truth, which he for ever falsifies, and of facts which he constantly distorts.

"March 29. ULTOR."

---

### 19. MEDICAL CONVERSAZIONES.

"EMOLLIT MORES NEC SINIT ESSE FEROS."

We are rejoiced beyond measure to perceive that, John Bull's "THIRD BRANCH OF THE FIFTH PAIR," has lately received a great accession of sensorial energy, and that the motility of certain muscles, supplied thereby, bids fair, in another season or two, to equal that of any of his neighbours on either side of the SEMPLON. It is quite refreshing to observe the *thaw* of ideas, (so long pent up and frozen in JOHNNY's encephalon) when we enter the rooms of Sir Astley Cooper—Mr. Pettigrew, or, last not least, the splendid saloon of the ROYAL COLLEGE of PHYSICIANS. It is not over a *cool* cup of tea or coffee, as over those maddening cups supplied by BACCHUS and CERES, that—

"Many a *long repented* word comes out."

No! these are the veritable "feasts of reason"—for in these rational CONVERSAZIONES, we renew (as it were) the "academic bowers" of classic times, and behold the PLATO's, the ARISTOTLE's, the LUCRETII, and the CELSI of the day, all eager in search of TRUTH—and earnestly inquiring "*de omnibus rebus et quibusdam aliis*," of which there are generally some rare and curious specimens on the tables.

In these social meetings, we hail the harbinger of better times—the olive branch of peace in medical society, interwoven with the laurel, and superseding or banishing the blood-stained dagger and murderous tommahawk, introduced of late, as the principal instruments of —strange to say—the HEALING ART!

That the ANARCHISTS—the SEPTEM-

brisers—the Triumviri* of the day, view, with great consternation, this "bond of union" spreading among all that is respectable in our profession, may be easily perceived by the pains which they take to turn it into ridicule. We confidently predict that these medical conversaziones will prove powerful auxiliaries to the re-action which is now taking place in the public mind against the most barbarous system of demoralising warfare that ever disgraced a liberal and learned profession. A momentary adumbration has darkened the medical atmosphere; but the natural good sense, love of justice, and generous character of Englishmen, will shortly dispel the cloud; and exhibit the adumbrators and mercenary instigators in all their native deformity.

We are glad to see that the College of Physicians has caught the rising spirit of the times, and thrown open its splendid library to these intellectual associations. The following is the card just issued from the College.

"The President and Fellows of the Royal College of Physicians will meet in the library of the College, for the purpose of reading medical papers, and holding medical conversation, at which they request ————'s attendance as often as it may be convenient to him, on all or any of the following evenings: viz. April 14—28; May 12—26; June 9—25. The meetings will be held from 9 till 11 o'clock."

Sincerely do we hope and anticipate, that these meetings will multiply—and that, in the course of another year, the metropolis will afford an opportunity, *every night in the week*, for medical men to come together—shake the hand of friendship—communicate their thoughts —rub off all asperities—drown petty jealousies—stifle animosities—and unite in the christian and philosophic labour of promoting harmony and good will among all members of the profession.

* "The first Triumvirate was in the hands of Cæsar, Pompey, and Crassus, who, at the expiration of their office, *kindled a civil war*. The second and last Triumvirate was under Augustus, Antony, and Lepidus, and, through them, *the Romans totally lost their liberty*. The Triumvirate was in full force at Rome for the space of about twelve years."

### 20. The English in Russia.

"But we far hence to distant climes must go,
O'er Caspia's deadly swamps, or Scythia's snow!"

An official invitation has lately been given by the Russian Government to the young medical men of this country, with the view of inducing them to enter the military and naval service of the Autocrat. It is by no means improbable that the crowded, distracted, and impoverished state of the profession, in this country, may drive many young men to embrace the offer of a living, however low the salary, beneath a foreign sky. Perhaps the situation of some medical men here could not be altered for the worse, by emigration to the Krimea, or even Siberia; and it is quite certain that the profession at large, in this kingdom, would be infinitely benefited by the exile, forced or spontaneous, of a few hundred of those turbulent souls who are disorganizing the very frame-work of medical society, and tearing up the foundations of social order and harmony in the profession. But we fear that this class of evil-doers is too well paid for their *services* here, to think of enlisting under a Russian banner, where the *knout* would very soon be their portion. It is therefore to put the honest but indigent medical youth on his guard, that we have noticed the invitation of the Russian Government. We must, in the first place, remark that the pay, not exceeding 45 pounds per annum for the first class, is quite inadequate for any thing like a comfortable subsistence, even in Russia. But the pay is much worse than it seems:— for, except in actual warfare, the rubles are paid in paper *which depreciates them more than one half!* In the Russian service the officers are allowed no rations, except in the field, and the pay, as above depreciated, is not sufficient to keep body and soul together. The rigorous climate of the northern, and the unhealthy atmosphere of the southern provinces, would destroy the lives of three fourths of our youth within the first ten years of their servitude. Add to this, the misery of living among semi-barbarians, where every scene around them forms a representation of portentous despotism on the one hand, and mournful prostration of mind on the other! For our own parts, we would rather serve in the ranks of a regiment, or before the mast of a ship of war, under the British crown, than be dignified with the rank of officer among the hordes of Calmucs or Cossacs in the dreary regions of Russia.

——— Quid terras alio calentes
Sole mutamus—patriæ quis exul
se quoque fugit?

---

21. LITHOTOMY.

The ears of the profession have been recently shocked by the REPORT of an operation for the stone at Guy's Hospital. If ever it was permitted for "a perturbed spirit" in human shape, to visit this earth, in the character of a REPORTER—then was there one at the elbow of the operator, on this occasion! The BEING who could indite, and send to the public newspapers such a report as that to which we allude—

"Is fit for treason, stratagems and spoils."

It is to be hoped, however, that, in the drama now acting on the professional stage, every incident will tend, directly or indirectly, to the completion of the final catastrophe—moral retribution! Whether a DIVINE POWER governs the current of events in this world—or, as some imagine, the PRINCE of DARKNESS may have a share in the direction, we leave for the determination of the philosopher. Of this, however, we feel assured—that the diabolical means which are now exerted to vilify, traduce, and assassinate the professional character of every public functionary, not belonging to a certain coterie, must excite—nay, has excited a sense of horror and disgust in the mind of every man who has a spark of honorable feeling yet lurking in his breast!

We have had but too many opportunities of witnessing—alas, of experiencing the untowardness of human nature; but still we are convinced that no member of the medical profession, unpossessed by an "evil spirit," could have constructed a report, breathing such revengeful aspirations as are conveyed in the document to which we allude. The ANIMUS in which the report is conceived and indited, will stamp the anonymous author, whoever he may be, as a disgrace to a liberal science. This last act so far outstrips any thing which we have been lately accustomed to see, that some exile from this upper world must have got loose from his prison-house, or the wandering spirit of a Nero, a Caligula, or a Heliogabalus must have been allowed, for some wise but inscrutable purpose, to experience a second incarnation!

Of all the operations which the intrepid hand of the surgeon has dared, LITHOTOMY is the most hazardous, the most occult, and, consequently, the most difficult. In all other operations, the eye assists the hand—but here, only a single sense can come into play, and a thousand circumstances may tend to deceive that sense, and baffle the adroitest hand and firmest nerve! The bye-stander, therefore, who could premeditatedly transmute a failure into a fault—and, with hellish ingenuity of language and manner, hold up the unfortunate operator as the murderer of his patient, must be totally divested of every particle of philanthropy or even humanity. But there is a fire that can never be slaked—there is a wound that can never heal—there is a worm that never dies—there is a remorse which the tomb cannot stifle. These, these must surely become the tyrant tenants of a bosom to which Golconda's gems, Peruvia's gold can never again restore

"The soul's calm sunshine."

The anonymous assassin of character may mix with the crowd, and plunge his concealed stiletto into the breast of his neighbour, unseen by mortal eye. But when he lays his head on his pillow, a thorn will be there—and the voice of conscience will ultimately cry—

"Sleep no more---Macbeth has murdered sleep."

Then will the pallid cheek and sunken eye betray the canker feeding on the soul, and years of remorse, and, it is to be hoped, of repentance, will succeed the frenzied enjoyment of a few short days of vindictive malice! Mean time, the evil is not yet at its height. Several years ago, we prophesied that the arm of the surgeon would be paralyzed, even in common and necessary operations, by the diabolical misrepresentations of malignant and unjust reporters. Our words have come true. No surgeon can now operate with safety, since every failure is blazoned forth in the newspapers, as an instance of ignorance or want of dexterity. The students, as well as the patients, will feel the consequences of this infernal system, unless some measure can be devised to check its ruthless career. The impression, however, is every day becoming more general, that the utter degradation of the medical character is at hand—and that medical men have been their own executioners in the encouragement which they have given to vituperative publications. The profession will learn wisdom in the school of adversity—but not till they are severely scourged for their folly.

### VARIETIES.

### 22. *Enlarged Livers and Spleens removed by Iodine.*

Three cases of this kind have recently been reported by Dr. Milligan, from the Royal Universal Infirmary for Children, which appear to prove the superiority of iodine over mercury in glandular and visceral tumours. In the first case, the child was five years of age, and presented a tumour occupying the right hypochondrium, extending anteriorly in a line with the linea alba, and inferiorly to the superior part of the hypogastric region. It was firm, colourless, pushed out the ribs considerably, and was attended occasionally with shooting pains. The tumour had been perceived growing for six months. The child was put on a course of the tincture of iodine, and in about two months and a half the tumour was quite gone.

In the second case, the child was under two years of age, and had an ague when admitted. There was a tumour in the region of the spleen, said to be of eight months growth. Mercury had been used without effect, and Dr. Milligan ordered the iodine in small doses. In little more than a month the swelling was gone. In the third case the tumour was lower than the region of the spleen; but it gave way to the tincture of iodine.

### 23. *Gangrene of the Lungs not fatal.*

Some pathologists of this country deny the possibility of recovery in cases where a portion of lung becomes disorganized in a gangrenous way, and where this disorganization is supposed to be indicated by the black fetid expectoration exhaling the odour or mortified parts. Laennec and others, on the Continent, maintain the possibility of such an occurrence, and we are inclined to side with such a man as Laennec, rather than with those who are opposed to him in this country. The following case was lately detailed by M. Laurent to the Parisian Academy of Medicine.

*Case.* Madam the Countes of L——, had experienced from youth several attacks of hæmoptysis. In a journey which she made to Versailles, in 1823, she was seized with acute pain in the right side of the chest, which yielded to the application of 24 leeches, but which still induced an indescribable malaise of two or three days' duration. On the 4th day, she was seized with a violent and convulsive cough, succeeded by an abundant expectoration of greenish matter mixed with a black substance, emitting a horrible gangrenous odour. This kind of expectoration continued a long time, and it was ten months before she perfectly recovered.—REVUE MED.

### 24. *Paralysis from Cubebs.*

Mr. Broughton has related a curious case of this kind, in the person of a young gentleman, who had been taking the above-mentioned medicine for about a fortnight, in the dose of two drachms thrice daily, for a gonorrhœa, and being otherwise in good health, and living quietly. A distortion of the mouth to one side, whenever he attempted to speak or smile, supervened—and the pulse became irregular, with some other symptoms of constitutional derangement. He was bled to half a pint, without any advantage. Then he was freely purged, with benefit, but at the end of three weeks the distortion had not entirely disappeared. Was this not an affection of the portio dura, and the result of cold caught in the side of the head, rather than the effect of cubebs?—MED. GAZ.

*N. B.* Fasciculus VII. which is printed by mistake at the top of page 1, should be Fasciculus I.
